# A multivariate twin study of the genetic association between present moment attention and subjective wellbeing

**DOI:** 10.1038/s41598-023-42810-x

**Published:** 2023-10-14

**Authors:** Kirk Warren Brown, Fazil Aliev, Thalia C. Eley, Danielle M. Dick, Chelsea Sawyers

**Affiliations:** 1 Carnegie Mellon University, Pittsburgh, USA; 2 Virginia Commonwealth University, Richmond, USA; 3 Rutgers Addiction Research Center, Rutgers University, Piscataway, USA; 4 King’s College London, London, UK

**Keywords:** Genetics, Psychology

## Abstract

Considerable evidence supports the role of present-moment attention, a central feature of mindfulness, in subjective wellbeing maintenance and enhancement. Yet it is not clear why such a relation exists. This study examined the genetic and environmental contributions of present-moment attention to subjective wellbeing. Consistent with the “generalist genes hypothesis” and prior evidence, we hypothesized that presence and subjective wellbeing would show a substantial genetic correlation and smaller environmental correlation. Using a large epidemiological sample of healthy 16-year-old twins in the United Kingdom (N = 1136 monozygotic (MZ) and dizygotic (DZ) twin pairs), genetic overlap was found between presence and the cognitive component of subjective wellbeing (life satisfaction), and to a lesser extent, the affective component of subjective wellbeing (operationalized as happiness). The non-shared environmental overlap between these constructs was substantial. This study provides the first evidence known to us showing that present-centered attention, a primary component of mindfulness, has both genetic and environmental overlap with subjective wellbeing. The findings have implications for understanding mechanisms by which presence is associated with positive emotions and life satisfaction, and suggest, pending additional research, that mindfulness-based interventions to enhance wellbeing may be best suited to those with a genetic propensity toward mindful presence.

## Introduction

Scientific and popular interest in wellbeing has flourished in the past two decades, spurred in part by evidence that wellbeing is associated with a range of positive biopsychosocial outcomes, including mental health, positive social relations, physical health, and longevity^1^. Along with interest in such effects has been a search for conditions supportive of wellbeing, and several theories have been proposed in this regard^2^, among them “mental state theories,” which highlight the role of states of attention in wellbeing. Flow theory^3^ and mindfulness theory (e.g.,^4^) are exemplars of such theories, and evidence supports the role of present-moment attention, or attention to current events and experiences, in subjective wellbeing maintenance and enhancement^5^. Yet it is not clear why such a relation exists, and the present study was designed to examine the genetic and environmental contributions of present-moment attention (“presence”) to subjective wellbeing.

### The phenotypic association between presence and subjective wellbeing

Subjective wellbeing is commonly considered a combination of cognitive evaluation, reflected in satisfaction with one’s life, and a predominance of positive over negative affective states over time. Presence is a central feature in various conceptualizations of mindfulness. It is reflected in a tendency to pay attention to what is currently taking place within and around oneself, rather than to be immersed in thoughts about the past or future, as in mind wandering, rumination, and other forms of cognitive time travel. Self-reported presence, as measured by instruments such as the Mindful Attention Awareness Scale^5^ and the *Act with Awareness* subscale of the Five Factor Mindfulness Inventory^6^ is phenotypically associated with higher subjective wellbeing^7^. Randomized trials of mindfulness training centrally feature instruction in focused, present-centered attention. They have shown pre-post intervention enhancements in subjective wellbeing, particularly increased positive affect and reduced negative affect (e.g.,^8,9^). Conversely, correlational and experimental research on mind-wandering has shown it to be associated with higher negative over positive affect^10^. Likewise, rumination has well-established associations with certain forms of negative affect (e.g., depression, anxiety^11^). Finally, both rumination and negative affect are moderately, inversely correlated with presence (e.g.,^5,6^).

Theoretical accounts have posited several psychological processes through which mindfulness enhances wellbeing, including attention regulation, emotion regulation, and altered self-perceptions (e.g.,^12^). But there is reason to hypothesize that mindfulness, and in particular its key component of presence, has genetic overlap with wellbeing. Previous research has found that both present-centered attention and subjective wellbeing have substantial genetic influence. For example, a twin study by Waszczuk et al.^13^ estimated that presence, as assessed with the Mindful Attention Awareness Scale, was 32% attributable to genetic influence. Other research has estimated 30–50% genetic influence on subjective wellbeing (e.g.,^14^). Recent large-sample genome-wide association studies have identified a number of polymorphisms associated with subjective wellbeing (e.g.,^15^). While no research has shown genetic links between presence and subjective wellbeing, Waszczuk et al.^13^ found genetic correlations of 0.52 and 0.53 between Mindful Attention Awareness Scale scores and depressive and anxiety sensitivity symptoms, respectively. Depressive symptoms in particular have been inversely correlated with subjective wellbeing (e.g.,^16^) and both phenotypes form part of a “wellbeing spectrum” with substantial genetic intercorrelations (e.g.,^15^).

Together this evidence concerning presence and wellbeing lends support to the “generalist genes hypothesis”^17^, which proposes that traits that covary commonly have similar genetic factors that account for their association, while environmental factors are typically smaller. In the case of presence and subjective wellbeing, however, there is an alternative hypothesis. Some activities, such as those that are intrinsically motivating and conducive to flow experiences rely on present-centered attention to the activity at hand and reliably induce states of wellbeing^3^. Additionally, contemplative practices such as meditation train attention and may result in higher wellbeing (e.g.,^2,18^). Thus, environmental influences may better explain the relation between presence and subjective wellbeing. By examining the contribution of both genetic and environmental factors in the association between presence and subjective wellbeing, the present investigation will promote our understanding of the biological and behavioral processes that link these traits^13^. Also, a better understanding of how individual differences in present-centered attention are related to subjective wellbeing can lead to more precise training to enhance wellbeing.

### The present study

The primary goal of this study was to examine the genetic overlap between presence and subjective wellbeing, using a large epidemiological sample of healthy 16-year-old twins in the United Kingdom. Consistent with the generalist genes hypothesis and prior evidence, we hypothesized that presence and subjective wellbeing would show a substantial genetic correlation and smaller environmental correlation. Our first aim was to test the phenotypic correlation between presence and subjective wellbeing. Our second aim was to examine the genetic and environmental influences on presence and subjective wellbeing variables individually. Third, we investigated what proportion of the expected phenotypic association between presence and subjective wellbeing was explained by genetic (relative to shared and nonshared environment) influences.

## Method

### Participants

Adolescent participants came from the population-representative Twins Early Development Study (TEDS), which comprises over 10,000 twin pairs born in England and Wales (see^19–21^ for recruitment details). The present study focuses on twins born between 1994 and 1996. Data for this study were collected in 2011, when the twins were approximately 16 years old on average. The parent study was approved by the Kings College London Research Ethics Committee (PNM/09/10-104). The study was performed in accordance with relevant guidelines and regulations. Informed consent was obtained from parents and all participating adolescents. Zygosity was established using parent-report questionnaires of physical similarity, a method estimated to be 95% accurate relative to DNA testing^22^. Where zygosity was unclear, DNA testing was conducted. Participants were excluded if they experienced severe pre- or perinatal complications, if they had a severe medical condition (e.g., chromosomal disorder, brain damage, autism, blindness), or if sex or zygosity were unclear. The sample for analyses consisted of 1136 monozygotic (MZ) and dizygotic (DZ) twin pairs (mean age = 16.89, SD = 0.23, range = 16.49–18.76); 429 MZ pairs (145 male pairs, 284 female pairs) and 707 DZ pairs (138 male pairs, 227 female pairs, 342 opposite-sex pairs).

### Measures

All measures were completed on paper and mailed in. Descriptive statistics on the study measures are presented in Table 1. Paired sample t-tests showed that the scores of first- and second-born twins did not differ on these measures, *p*s > 0.197.

**Table 1:** Descriptive statistics on the primary study variables by zygosity and gender.

	Mean (SD)	Range
MZ males		
Presence	4.35 (0.88)	2.60–6.00
Happiness	5.08 (1.03)	1.50–7.00
Life satisfaction	5.80 (1.01)	2.00–7.00
Subjective wellbeing	5.44 (0.91)	2.58–7.00
MZ females		
Presence	4.20 (0.90)	1.40–6.00
Happiness	5.16 (1.00)	1.00–7.00
Life satisfaction	5.71 (1.03)	1.17–7.00
Subjective wellbeing	5.45 (0.91)	2.13–7.00
DZ males		
Presence	4.29 (0.90)	1.80–6.00
Happiness	5.08 (1.09)	1.00–7.00
Life satisfaction	5.76 (1.01)	1.00–7.00
Subjective wellbeing	5.44 (0.99)	2.38–7.00
DZ females		
Presence	4.17 (0.88)	1.50–6.00
Happiness	4.98 (1.05)	1.50–7.00
Life satisfaction	5.51 (1.08)	2.00–7.00
Subjective wellbeing	5.21 (0.99)	2.25–7.00
DZ opposite sex		
Presence	4.17 (0.82)	1.80–6.00
Happiness	5.04 (1.03)	1.00–7.00
Life satisfaction	5.64 (1.02)	1.33–7.00
Subjective wellbeing	5.40 (0.92)	1.67–7.00

Subjective well-being is calculated as a mean of the happiness and life satisfaction variables. SD, Standard deviation.

#### Presence

Present-centered attention was measured using a brief (5-item), item response theory-validated version^23^ of the Mindful Attention Awareness Scale^5^. The items focus on inattentiveness to current events and experiences (e.g., “I find myself doing things without paying attention”) with response options ranging from “almost always” to “almost never.” Previous research has shown that the scale items correlate highly with items phrased oppositely, as attentiveness^5^. This lends credibility to the scale as a measure of presence, rather than simply a lack thereof. Responses were averaged to provide trait presence scores; higher scores reflect higher presence. Sample Cronbach’s alpha was acceptably high (α = 0.75).

#### Subjective wellbeing

Two validated scales were administered that together assessed subjective wellbeing. The 4-item Subjective Happiness Scale^24^ assesses subjective perceptions of general happiness (example item: “In general I consider myself:” with response options ranging from “not a very happy person” to “a very happy person.” Responses were averaged to provide a composite score; higher scores reflect greater happiness (sample α = 82). The 5-item Brief Multidimensional Student Life Satisfaction Scale^25^ assesses satisfaction with one’s life in five domains: family, school, friends, self, and living environment (example item: “I would describe my satisfaction with my family life as:” with response options ranging from “terrible” to “delighted”^26^). Responses were summed to create a total life satisfaction score (sample α = 85). Large-scale studies report that subjective wellbeing is a latent factor that comprises affect and life satisfaction in both adults^26,27^ and children^28^. To assess subjective wellbeing at a global level in supplementary analyses, Subjective Happiness Scale scores (“happiness”) and Brief Multidimensional Student Life Satisfaction Scale scores (“life satisfaction”) were averaged together, as they used the same 1–7 Likert scaling.

### Statistical analyses

The study analyses were preregistered on OSF (https://osf.io/6fd7m). Analyses were based on the twin method^29^, which compares the degree of similarity between MZ twins (sharing 100% of their genetic variations) and DZ twins (sharing, on average, 50% of their genetic variations). These relative differences in within-pair correlations allow estimations of the influences caused by additive genetic, shared environmental, and nonshared environmental influences on a trait or the covariation between traits. Where correlations are higher for MZ twins than for DZ twins, genetic influences are inferred to be acting (*A*). Within-pair similarity that is not due to genetic factors is accounted for by shared environmental influences (*C*), which contribute to the resemblance between family members. *C* is evident when DZ correlations are more than half MZ correlations. Nonshared environment (*E*) accounts for factors that create individual differences between siblings. These are estimated from within-pair differences between MZ twins. *E* also includes measurement error.

Twin model fitting was performed in OpenMx^30^ within the *R* package (www.R-project.org) to examine Aims 1–3. Aim 1 concerned the phenotypic correlations (*r*_*ph*_) between presence and subjective wellbeing (happiness and life satisfaction separately). Aims 2 and 3 concerned the genetic and environmental bases of these associations. A constrained saturated model was first used to calculate twin and cross-twin cross-trait correlations. Twin correlations are within-twin pair, within-trait correlations; specifically, correlations within MZ pairs (*r*_MZ_) and within DZ pairs (*r*_DZ_) were computed for presence, happiness, and life satisfaction separately. Cross-twin cross-trait correlations were computed by correlating a trait in one twin with another trait in the co-twin (e.g., presence with happiness). Twin and cross-twin cross-trait correlations permit a first look at how individual differences and associations between them are due to genetic (A), shared environmental (C), and nonshared environmental (E) factors.

**Figure 1:**
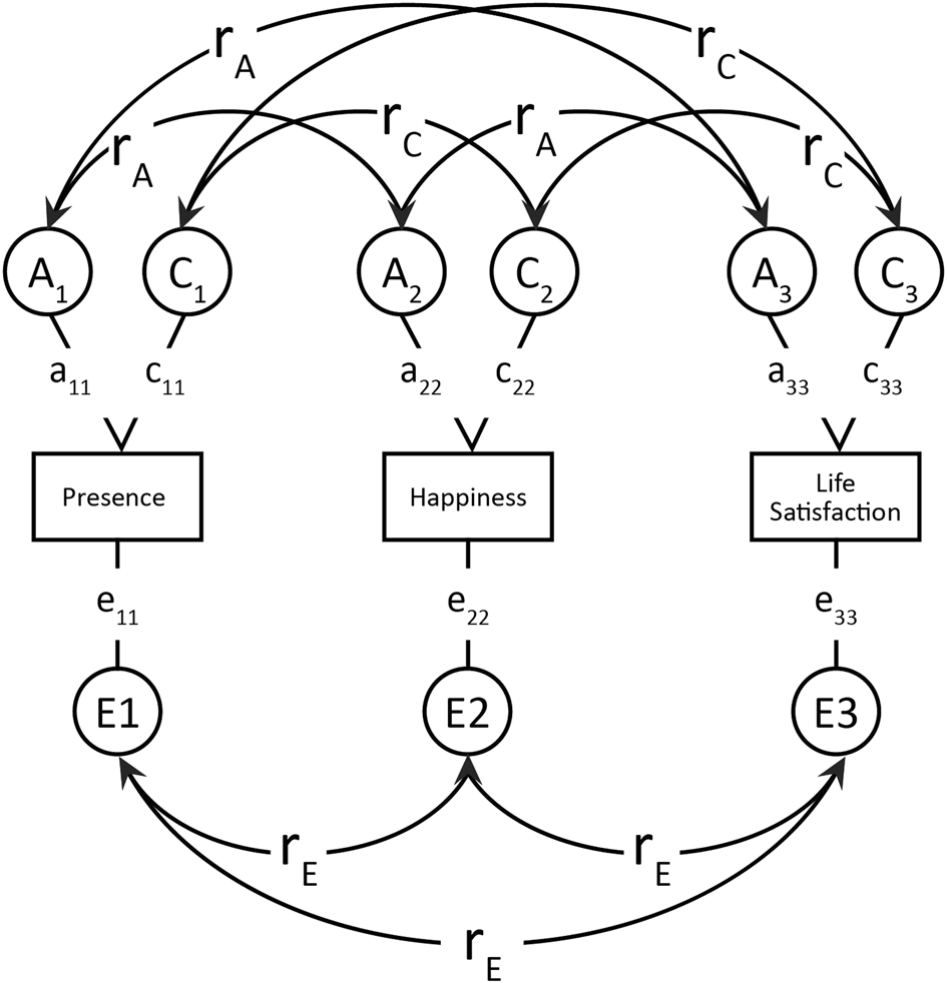
Trivariate ACE model. *Note* Rectangles refer to the variance of observed variables. Circles refer to latent genetic (*A*), shared environmental (*C*) and non-shared environmental (*E*) factors. All latent variables have a variance = 1. Curved double-headed arrows refer to genetic and environmental correlations (*r*_*A*_, *r*_*C*_, *r*_*E*_).

Building on the initial results revealed by twin and cross-twin cross-trait correlations, a multivariate correlated factors model (Fig. 1) was fitted using a direct variance estimation approach^31^ to address aims 2 and 3 of this study. Specifically, *r*_*ph*_*,* A, C, and E influences on presence and the subjective wellbeing variables were estimated, as well as the genetic and environmental correlations between these variables (*r*_*A*_*, r*_*C*_*, r*_*E*_). These latter correlations indicate the degree of genetic and (shared, nonshared) environmental overlap between two traits. The proportion of the phenotypic correlations (*r*_*ph*_) due to genetic or environmental factors was also estimated. For example, using the nomenclature shown in Fig. 1, the proportion of *r*_*ph*_ between presence and life satisfaction can be estimated as *r*_A_ $\times$√*a*_11_ $\times$√*a*_22_, divided by *r*_*ph*_. All variables were normally distributed so no transformations were applied.

Following standard procedures, the variables were regressed for sex^32^. All models were fitted using full information maximum likelihood to account for missing data. The core fit statistic was minus twice the log likelihood (− 2LL) of the observations. This is not an overall measure of fit, but provides a relative measure of fit, since differences in − 2LL between models are distributed as *χ*^2^. Therefore, to examine the overall fit of the genetic model we compared the − 2LL to that of a saturated model (one which fully describes data using the maximum number of free parameters, estimating variances, covariances, and means for the raw data to geta baseline index of fit). The fit of each submodel was assessed by *χ*^2^ difference tests, Akaike’s and Bayesian’s information criterion (AIC = *χ*^2^ − 2*df*, BIC = *χ*^2^ − *k*ln(*n*)) with lower *χ*^2^ values, and more negative AIC and BIC values suggesting a better fit. If the difference between the AIC of two models was less than 10, the more parsimonious model was selected^33^. For all analyses, we also compared models with fewer parameters to the full *A*, *C*, and *E* correlated factors solution. To assess the precision of parameter estimates, 95% confidence intervals (CIs) were obtained. CIs that include zero indicate that an estimate is nonsignificant. CIs that do not overlap indicate that two estimates differ significantly.

### Ethics approval

The Institutional Review Board of Kings College London approved all procedures performed in this study involving human participants.

### Informed consent

All participants provided informed consent prior to participation in the study.

## Results

### Phenotypic, twin, and cross-twin cross-trait correlations

Presence was related to higher subjective wellbeing (Table 2). Specifically, presence was modestly but significantly correlated with happiness and life satisfaction (*r*_*p*_ = 0.18 and 0.24 respectively). The difference in the magnitude of these correlations was significant, *p* = 0.016. The MZ twin correlations were larger than the DZ correlations (Table 2) but less than 1, indicating additive genetic (A) and nonshared environmental (E) influences on presence and both subjective wellbeing variables (happiness, life satisfaction; parallel results for overall subjective wellbeing are presented in supplementary Table 1). Table 2 also shows that the MZ twin correlation between presence and happiness was more than double the DZ correlation, suggesting: (a) a lack of shared environmental influences (*A, E* rather than *A, C, E*); and (b) that genetic influence on this trait is best interpreted as additive (sum of individual alleles at all loci that influence the trait). The MZ twin correlations between presence and both life satisfaction (Table 2) and overall subjective wellbeing (supplementary Table 1) were less than double those for DZ twins (Table 2) indicating some shared environmental (C) influence.

**Table 2:** Phenotypic, twin, and cross-twin cross-trait (CTCT) correlations between presence and subjective well-being traits.

	Presence	Happiness	Life satisfaction
Phenotypic correlations			
Presence	–		
Happiness	0.18 (0.14–0.22)	–	
Life satisfaction	0.24 (0.20–0.28)	0.63 (0.60–0.65)	–
MZ and DZ twin correlations			
MZ	0.37 (0.31–0.42)	0.39 (0.33–0.45)	0.55 (0.50–0.60)
DZ	0.16 (0.10–0.21)	0.26 (0.21–0.31)	0.32 (0.27–0.36)
MZ (below diagonal) and DZ (above diagonal) CTCT correlations			
Presence	–	0.09 (0.02–0.17)	0.10 (0.02–0.17)
Happiness	0.08 (− 0.01–0.17)	–	0.23 (0.16–0.30)
Life satisfaction	0.16 (0.06–0.25)	0.37 (0.28–0.45)	–

MZ, Monozygotic; DZ, Dizygotic; CTCT, Cross-twin cross-trait. 95% Confidence intervals (CIs) are presented in brackets.

The cross-twin cross-trait correlation between presence and life satisfaction was larger for MZ twins than for DZ twins (Table 2), which suggests a role for additive genetics factors (A) in the association between these two variables and a smaller role for shared environmental influences in the association between these traits. However, the size of the cross-twin cross-trait correlation between presence and happiness did not differ between MZ and DZ twins (Table 2). Likewise, the magnitude of the cross-twin cross-trait correlation between presence and overall subjective wellbeing was only slightly larger for MZ than for DZ twins (supplementary Table 1). These results suggest no additive genetics factors in the association between these variables, and instead suggest some role for shared environmental (C) influences in the association between them. The MZ cross-twin cross-trait correlations between presence and the two subjective wellbeing variables were considerably smaller than the phenotypic correlations (Table 2), suggesting that nonshared environments (E) also played a meaningful role in explaining the association between presence and the subjective wellbeing variables. Parallel results for overall subjective wellbeing are in supplementary Table 1.

### ACE model results

Table 3 shows that the ACE model (correlated factors solution; see Fig. 1) fit equally well as the saturated model, indicating a good fit to the data. The AE model showed the best fit to the data, based on the nonsignificant χ^2^ difference in fit from the ACE model and its lowest AIC and BIC values. The CE and E models showed significantly poorer fit to the data (Table 3). There were no effects of sex in model fit (see supplementary Table 2). Table 4 shows that significant genetic influence was found for presence (A = 34%), SHS happiness (A = 42%), and life satisfaction (A = 57%). The rest of the variance was due to nonshared environmental influences for all three variables (Table 4). Inspection of the genetic and environmental correlations between presence and wellbeing traits (*r*_*A*_ and *r*_*E*_; Table 4) indicates that both etiological correlations were significant, though the genetic correlations were somewhat larger. The genetic correlation between presence and life satisfaction (*r*_*A*_ = 0.36) was significantly larger than that between presence and happiness (*r*_*A*_ = 0.22), *p* < 0.001. Estimates of the proportion of *r*_*ph*_ due to A and E indicate that shared genetic influences explained approximately half the phenotypic correlation of presence with happiness (% *r*_*ph*_ due to A = 48%, E = 52%; Table 4). Shared genetic influences explained more than 2/3 of the phenotypic correlation between presence and life satisfaction (% *r*_*ph*_ due to A = 68%, E = 32%; Table 4). Supplementary Table 3 shows parallel results for presence and overall subjective wellbeing.

**Table 3:** Multivariate model fit statistics.

	− 2LL	*Df*	*χ*^2^	*Δdf*	*p*	AIC	Size-adjusted BIC
(a) Comparison to saturated model							
Saturated model	17,485.59	6682				4121.59	17,693.97
Correlated factors solution (ACE)	17,502.88	6717	17.29	35	0.99	4068.88	17,576.20
(b) Comparison to correlated factors solution (ACE)							
Correlated factors solution (AE)	17,502.80	6723	5.10	6	0.53	4066.80	17,572.26
Correlated factors solution (CE)	17,543.30	6723	45.59	6	< 0.05	4107.30	17,612.76
Correlated factors solution (*E*)	17,840.17	6729	342.46	12	< .0.05	4392.17	17,886.47

− 2LL, Minus twice the log likelihood; *df* , Degrees of freedom; *p*, Probability; AIC, Akaike’s information criterion; BIC, Bayesian’s information criterion. The best fitting model (correlated factors solution, AE) was selected based on the principle of parsimony and lowest AIC and BIC value.

**Table 4:** Standardized genetic and environmental parameter estimates (on diagonals), genetic and environmental correlations (below diagonals), and proportions of phenotypic correlations due to genetic and environmental factors (above diagonals).

	Presence	Happiness	Life satisfaction
*A* estimates			
Presence	**0.34 (0.27–0.41)**	0.48 (0.18–0.74)	0.68 (0.48–0.86)
Happiness	0.22 (0.08–0.32)	**0.42 (0.35–0.49)**	0.62 (0.53–0.71)
Life satisfaction	0.36 (0.25–0.48)	0.80 (0.74–0.86)	**0.57 (0.50–0.62)**
*E* estimates			
Presence	**0.65 (0.58–0.73)**	0.52 (0.26–0.81)	0.32 (0.14–0.50)
Happiness	0.15 (0.07–0.22)	**0.58 (0.51–0.65)**	0.38 (0.30–0.44)
Life satisfaction	0.14 (0.06–0.22)	0.47 (0.41–0.53)	**0.43 (0.38–0.49)**

Results from a trivariate ACE model (see Fig. 1). 95% confidence intervals (CIs) are in parentheses. Shared environmental *(C)* parameter estimates are not included as they were non-significant in the multivariate model fit statistics (see Table 2). *A* Additive genetic parameters; *E* Nonshared environmental parameters.

## Discussion

Over the past several decades there has been a burgeoning interest in mindfulness, of which present-centered attention (presence) is theorized to be a key component^34^. Numerous studies have found that presence is associated with subjective wellbeing (affect and life satisfaction) but unknown are the bases for this association. The current study is the first known to us to examine the genetic and environmental etiologies of the association between self-reported dispositional presence and subjective wellbeing. We hypothesized that presence and wellbeing would show a substantial genetic correlation and smaller environmental correlation, in accord with the generalist genes hypothesis^17^ as well as prior evidence. Genetic overlap was found between presence and the cognitive component of subjective wellbeing (life satisfaction), and to a lesser extent, the affective component of subjective wellbeing (operationalized here as happiness). The environmental overlap between these constructs was substantial, however.

We first showed that, as in other research over the years, there was a phenotypic association between presence and the subjective wellbeing variables (happiness, life satisfaction, and their combination). These correlations were smaller than those seen in previous studies, but most prior research on this topic has been conducted with adults (e.g.,^5^) rather than with adolescents. Also, this study used a brief version of the Mindful Attention Awareness Scale. While the internal consistency of the measure was adequate, it is possible that higher phenotypic correlations might be obtained with the full version of the scale. For example, Brown et al.^35^ found higher correlations between the full Mindful Attention Awareness Scale and both affect and life satisfaction in a large sample of normative adolescents (mean age = 16.73 years). Thus the results obtained here concerning genetic and environmental influences on presence, subjective wellbeing variables, and their associations might be somewhat measure-dependent.

The twin models showed both genetic and environmental overlap between presence and subjective wellbeing variables. Thus, some genes associated with presence also appear to play a role in subjective wellbeing. The generalist genes hypothesis^17^ argues that phenotypic covariance is commonly attributable to similar genetic factors, while environmental factors are typically smaller. The present results showed genetic and environmental factors each explained approximately half of the phenotypic covariation between presence and subjective wellbeing variables. These results have implications for better understanding the oft-observed phenotypic association between presence and subjective wellbeing. Both mindfulness^34^ and wellbeing^2^ have been considered, in part, inherent dispositions, but also trainable skills, as shown by research on mindfulness^18^ and well-being^36^.

There is growing interest in understanding the mechanisms that link presence and wellbeing. The shared genetic etiology of these two traits begs a question concerning the neurobiological mechanisms linking them. This topic has not been investigated directly, but research has found links between presence, as measured by the Mindful Attention Awareness Scale and other, related measures, and neural markers of emotion regulation, assessed both through encephalography (e.g.,^37–39^) and functional magnetic resonance imaging (e.g.,^40–44^; see^45^ for review). Emotion regulation, in turn, is an important underpinning for subjective wellbeing^46^. Mind-wandering also links presence^47^ and wellbeing^10^, and mind-wandering has been associated with default mode network activity in the brain^48^. If both presence and subjective wellbeing are associated with reduced mind-wandering and default mode network activity in the brain, then these psychological and neural factors may also, along with emotion regulation, represent mechanisms of the genetic link between presence and wellbeing.

Consistent with previous reports using the TEDS database^13,49^, this study found evidence of substantial genetic influence on presence, as assessed by the Mindful Attention Awareness Scale. To date, research on the etiology of presence has explored the role of socialization in the development of this trait^50^. This study found considerable evidence of environmental influences on present-centered attention, yet most of this was due to non-shared factors, suggesting that social or other influences may be rooted in differential treatment of children in the family home or rooted in extra-familial factors (e.g., peer influences; unique life events), as appears to be true for SWB^14^. The question of how mindfulness or presence develops is, we argue, an important one to better understand how to foster this adaptive disposition.

### Limitations and future directions

This study had a large sample, permitting genetically informed analyses. It was limited in several ways however. First, the sample was composed largely of White participants, limiting the conclusions to this population. The results may also not generalize to adult populations since genetic and environmental influences on psychological traits can change with age. Research is needed to examine whether the results found here concerning the genetics of present-centered attention and the genetic and nonshared environmental origins of the associations between presence and wellbeing traits extend to non-White adolescent populations and to diverse populations of adults. Second, the reliance on self-report measures introduced the possibility of shared method variance, which may have inflated parameter estimates. In particular, method variance, if it existed, would inflate non-shared environmental correlations (rather than genetic correlations). If different methods to measure the study variables were to be used, the non-shared environmental correlations might be smaller than those reported here.

A third limitation is that the study cannot inform about mindfulness, since present-centered attention is considered an element of mindfulness, not the entire phenomenon^51^. However recent research using network analysis indicated that two of the most commonly used mindfulness scales (Mindful Attention Awareness Scale; Five Factor Mindfulness Questionnaire) actually reflect more well-established constructs concerning executive attention and other psychological phenomena^51^. Thus the simpler concept of presence may represent a more appropriate level of investigation of day-to-day attention, which mindfulness scales in part purport to assess. Additionally, the Mindful Attention Awareness Scale has been shown to be associated with neural activation and functional connectivity in and between attention regions of the brain that have been associated with mindfulness-based interventions as well^45^. This lends credibility to the use of the Mindful Attention Awareness Scale to assess a component of mindfulness.

A fourth limitation is inherent to twin (and family) studies of genetic influence. Studies using twin designs have assumptions and limitations concerning equal environments, gene-environment correlation, and gene x environment interaction (GxE) effects. The implication is that this study’s findings concerning genetic influences on presence, subjective wellbeing variables, and their associations could include interactions between genes and shared environments^49^.

Finally, it is unclear why the genetic presence—life satisfaction overlap was considerably larger than that between presence and happiness; we speculate that the two former measures tap quite stable characteristics relative to the happiness measure, which may be more subject to circumstantial influences.

In conclusion, this study provides the first evidence known to us showing that a component of mindfulness, namely present-centered attention, has both genetic and non-shared environmental overlap with subjective wellbeing. This has implications for our understanding of the mechanisms by which presence is associated with positive emotions and life satisfaction. On the environmental side of the equation, it could be that positive or negative life experiences might impact both presence and wellbeing. On the genetic side, consideration of genetic influences may inform mindfulness-based interventions to enhance wellbeing; such interventions may be best suited to those with an inherent propensity toward mindful presence. Yet is highly unlikely that that such interventions will *only* benefit those with a particular genetic makeup; while genes may be influential, they are not typically deterministic.

Among next steps in this line of work, research is needed to identify specific genes associated with presence. Genome-wide association studies (GWAS), have not been conducted to examine associations between measured genetic variants and present-centered attention, or mindfulness, but GWAS has been performed to identify genes associated with subjective wellbeing^15,52^. The derived polygenic scores, wherein individual genetic effects are summed together, could be used to more closely examine genetic overlap between wellbeing and presence phenotypes in future research.

### Supplementary Information

Supplementary Tables.

## Data Availability

The data that support the findings of this study came from the Twins Early Development Study (TEDS). Eligible researchers can apply for access to the TEDS data: https://www.teds.ac.uk/researchers/teds-data-access-policy.
