# Supplementary material for: A multivariate twin study of the genetic association between present moment attention and subjective wellbeing
Source: Sci Rep. 2023 Oct 14;13:17456. doi: 10.1038/s41598-023-42810-x (PMC10576771; doi:10.1038/s41598-023-42810-x)
Supplement: Supplementary file 1 — Supplementary Tables. [file 41598_2023_42810_MOESM1_ESM.pdf]

Supplementary Tables for

A multivariate twin study of the genetic association between present moment attention and subjective wellbeing

Kirk Warren Brown, Fazil Aliev, Thalia C. Eley, Danielle M. Dick, Chelsea Sawyers

Supplementary table 1. Phenotypic, twin, and cross-twin cross-trait (CTCT) correlations between presence and subjective wellbeing

	Presence	Subjective well-being
Phenotypic correlations		
Presence	—	
Subjective wellbeing	0.23 (0.19–0.27)	—
MZ and DZ twin correlations		
MZ	0.37 (0.31–0.42)	0.52 (0.47–0.57)
DZ	0.16 (0.10–0.21)	0.32 (0.27–0.36)
MZ (below diagonal) and DZ above diagonal) CTCT correlations		
Presence	—	0.11 (0.04–0.18)
Subjective wellbeing	0.13 (0.03–0.22)	—

Notes: MZ = monozygotic, DZ = dizygotic; CTCT = cross-twin cross-trait. 95% Confidence intervals (CIs) are presented in brackets.

Supplementary Table 2. Sex limitation multivariate model fit statistics

	−2LL	*df*	χ^2	Δdf	*p*	AIC	Size-adjusted BIC
(a) Comparison to saturated model							
Saturated model	6264.23	2219	-	-	-	6314.23	6360.70
Qualitative (Ra) & quantitative sex differences	6280.64	2235	18.64	16	.28	6298.64	6315.36
(b) Comparison to General non-scalar sex limitation							
Qualitative (Rc) & quantitative sex differences	6280.64	2235	<.001	0	1	6298.64	6315.36
Quantitative non-scalar sex differences	6280.86	2236	0.22	1	.63	6296.86	6311.74
No Sex differences ACE model	6285.26	2239	4.62	4	.32	6295.26	6304.55

Notes: -2LL = minus twice the log likelihood; *df* = degrees of freedom; *p* = probability; AIC = Akaike’s information criterion; BIC = Bayesian’s information criterion. The best fitting model (No sex differences ACE) was selected based on the principle of parsimony and lowest AIC and BIC value.

Supplementary table 3. Genetic and environmental parameter estimates (on diagonals), genetic and environmental correlations (below diagonals), and proportions of phenotypic correlations due to genetic and environmental factors (above diagonals)

	Presence	Subjective wellbeing
***A* estimates**		
Presence	**0.38 (0.16–0.59)**	0.59 (0.37–0.77)
Subjective wellbeing	0.31 (0.19–0.43)	**0.43 (0.25–0.60)**
***C* estimates**		
Presence	**-0.03 (-0.19–0.13)**	
Subjective wellbeing		**0.10 (-0.04–0.24)**
***E* estimates**		
Presence	**0.65 (0.57–0.73)**	0.42 (0.23–0.61)
Subjective wellbeing	0.17 (0.10–0.25)	**0.47 (0.41–0.53)**

Note. Results from a bivariate ACE model. *A* = additive genetic parameters; *C* = shared environmental parameters; *E* = nonshared environmental parameters. 95% confidence intervals (CIs) are in parentheses. The wide CIs around shared environmental *(C)* parameter estimates for both variables suggest they cannot be reliably estimated. In the multivariate model fit statistics (not shown), *C* is estimated at zero.
